# Endoscopic nipple-sparing mastectomy with immediate DIEP flap reconstruction following neoadjuvant chemotherapy for multifocal HER2-positive breast cancer: a case report

**DOI:** 10.1093/jscr/rjag344

**Published:** 2026-05-21

**Authors:** Pon Jeeva Mathan, Keshavarajan Gandhirajan, Jagadesh Chandra Bose, Sivakumar Arumugam, Pradheep Manivasagam, Sirajudeen Kamaldeen

**Affiliations:** Department of Surgical Oncology, Dr. Rela Institute & Medical Centre, 7, CLC Works Road, Nagappa Nagar, Chromepet, Chennai, Tamil Nadu 600044, India; Department of Surgical Oncology, Dr. Rela Institute & Medical Centre, 7, CLC Works Road, Nagappa Nagar, Chromepet, Chennai, Tamil Nadu 600044, India; Department of Surgical Oncology, Dr. Rela Institute & Medical Centre, 7, CLC Works Road, Nagappa Nagar, Chromepet, Chennai, Tamil Nadu 600044, India; Department of Surgical Oncology, Dr. Rela Institute & Medical Centre, 7, CLC Works Road, Nagappa Nagar, Chromepet, Chennai, Tamil Nadu 600044, India; Desire Aesthetics, No. 3, VOC Nagar 1st Street, Anna Nagar East, Chennai, Tamil Nadu, India; Department of Surgical Oncology, Dr. Rela Institute & Medical Centre, 7, CLC Works Road, Nagappa Nagar, Chromepet, Chennai, Tamil Nadu 600044, India; Desire Aesthetics, No. 3, VOC Nagar 1st Street, Anna Nagar East, Chennai, Tamil Nadu, India; Department of Surgical Oncology, Dr. Rela Institute & Medical Centre, 7, CLC Works Road, Nagappa Nagar, Chromepet, Chennai, Tamil Nadu 600044, India

**Keywords:** endoscopic nipple-sparing mastectomy, DIEP flap reconstruction, neoadjuvant chemotherapy, HER2-positive breast cancer, autologous breast reconstruction, minimally invasive breast surgery

## Abstract

Endoscopic nipple-sparing mastectomy (E-NSM) is a minimally invasive approach that enables oncologically sound breast resection whilst minimizing visible scarring. Its application following neoadjuvant chemotherapy (NACT), particularly in combination with immediate autologous reconstruction using a deep inferior epigastric artery perforator (DIEP) flap, remains infrequently reported. We describe a 38-year-old woman with multifocal HER2-positive invasive ductal carcinoma of the right breast who achieved an excellent clinical and radiological response to HER2-directed neoadjuvant chemotherapy. She subsequently underwent E-NSM through a single axillary incision with intraoperative retroareolar frozen section assessment, followed by immediate DIEP flap reconstruction. Final histopathology demonstrated a pathological complete response (ypT0 ypN0; RCB-0). The postoperative course was uneventful, with full nipple–areolar complex viability and a satisfactory aesthetic outcome. This case demonstrates the feasibility and safety of this combined approach in carefully selected patients.

## Introduction

Preserving breast aesthetics without compromising oncologic outcomes has become a central objective in modern breast cancer surgery. Nipple-sparing mastectomy has improved cosmetic satisfaction; however, conventional open approaches often leave visible scars on the breast mound. Endoscopic techniques enable remote incisions, enhanced visualization, and precise dissection, thereby reducing external scarring [1–3]. Endoscopic nipple-sparing mastectomy has been increasingly adopted in parts of East Asia, with encouraging oncologic and cosmetic results [4, 5]. Nevertheless, reports describing its use in combination with immediate autologous reconstruction particularly after neoadjuvant chemotherapy remain limited. Autologous reconstruction with a deep inferior epigastric artery perforator (DIEP) flap offers long-term durability and avoids implant-related complications [6, 7]. We report a case of endoscopic nipple-sparing mastectomy (E-NSM) with immediate DIEP flap reconstruction following neoadjuvant chemotherapy for multifocal HER2-positive breast cancer. This case is reported in accordance with the SCARE 2025 guidelines [8].

## Case presentation

### Patient information

A 38-year-old woman presented with a progressively enlarging lump in the upper outer quadrant of the right breast. She had no significant comorbidities, no prior breast surgery, and no family history of breast cancer. She did not smoke or consume alcohol.

### Clinical findings

Physical examination revealed a firm, mobile mass measuring ~3 × 3 cm in the right breast. The overlying skin and nipple areolar complex were unremarkable. No palpable axillary lymphadenopathy was noted.

### Diagnostic assessment

Breast imaging with mammography and ultrasonography demonstrated multifocal suspicious lesions. Positron emission tomography computed tomography confirmed metabolically active disease confined to the breast without distant metastasis. Core needle biopsy revealed grade II invasive ductal carcinoma with HER2 overexpression and a high proliferative index [9].

### Therapeutic intervention

**Figure 1:**
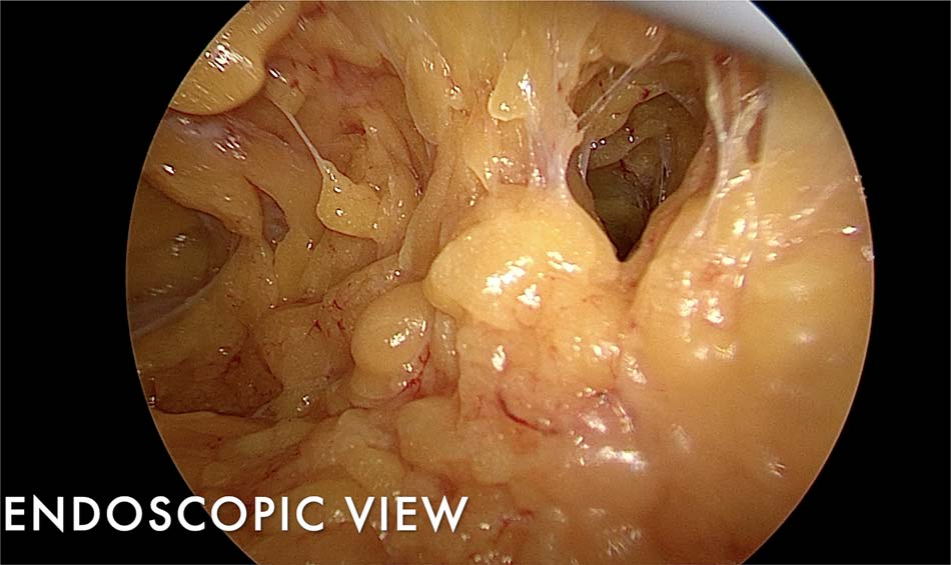
Endoscopic view during nipple-sparing mastectomy dissection demonstrating uniform subcutaneous flap thickness with clear delineation of Cooper’s ligaments, allowing precise glandular separation whilst preserving skin and nipple–areolar complex vascularity.

**Figure 2:**
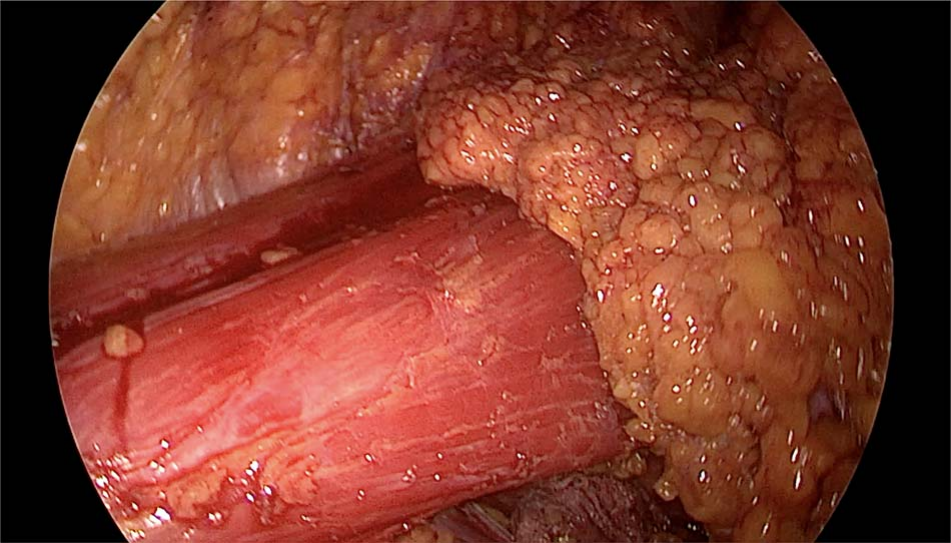
Endoscopic identification of the pectoralis major muscle during subfascial dissection, showing exposed muscle fibres after completion of retromammary dissection and preservation of chest wall integrity.

**Figure 3:**
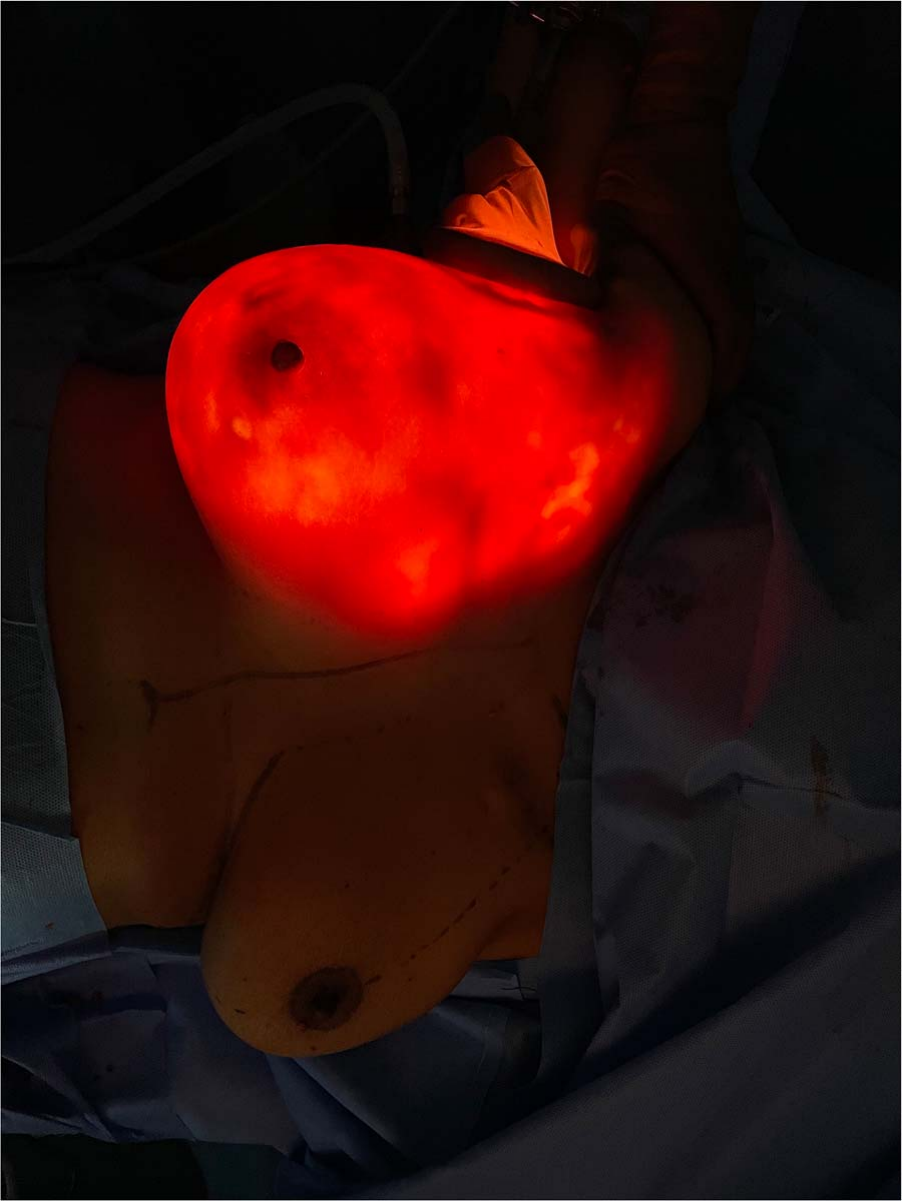
Intraoperative transillumination assessment following endoscopic nipple-sparing mastectomy demonstrating uniform light transmission through the mastectomy flap and well-perfused nipple–areolar complex.

The mastectomy was performed endoscopically through a single axillary incision. Tumescent infiltration and carbon dioxide insufflation facilitated elevation of the mastectomy flap under magnified endoscopic vision, enabling precise dissection within the subcutaneous plane whilst maintaining uniform flap thickness (Fig. 1). Subfascial dissection allowed clear identification of the pectoralis major muscle and safe separation of the breast gland from the chest wall (Fig. 2). Intraoperative transillumination was used to assess flap thickness and vascularity, confirming uniform perfusion of the mastectomy skin flap and nipple–areolar complex (Fig. 3).

A retroareolar frozen section was obtained intraoperatively and confirmed the absence of malignant involvement, permitting nipple preservation. Following completion of the mastectomy and axillary clearance, immediate reconstruction was undertaken using a DIEP flap harvested with preoperative perforator mapping. Microvascular anastomosis to regional recipient vessels was performed with satisfactory flap perfusion [6, 10].

### Follow-up and outcomes

**Figure 4:**
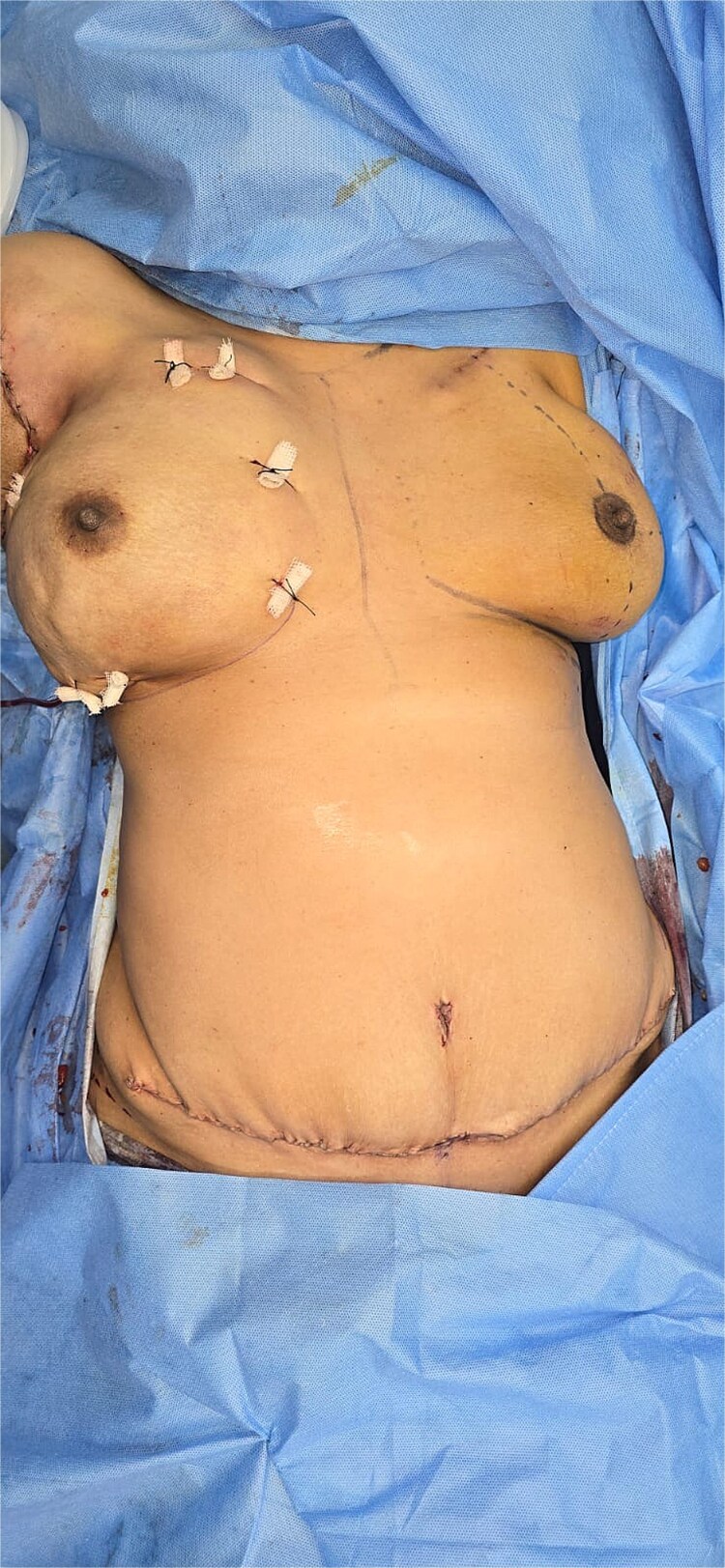
Final operative outcome showing the reconstructed breast and closed abdominal donor site following immediate DIEP flap reconstruction, with healthy mastectomy skin flap perfusion and meticulous donor-site closure.

**Figure 5:**
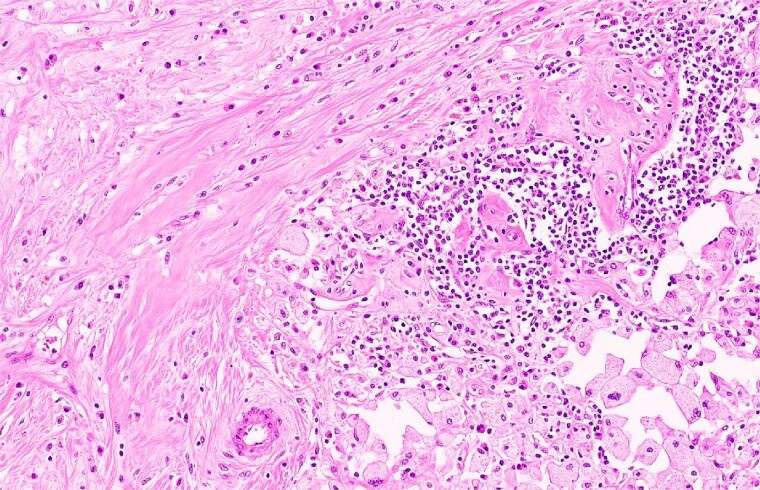
Histopathological examination (H&E stain) demonstrating tumour bed fibrosis with inflammatory infiltrate and hemosiderin-laden macrophages, with no residual invasive or in situ carcinoma.

The postoperative course was uneventful. The DIEP flap remained well perfused, and the nipple–areolar complex was fully viable. The final operative outcome demonstrated satisfactory breast contour with minimal donor-site morbidity (Fig. 4). Final histopathological examination showed no residual invasive or in situ carcinoma within the breast specimen, with tumour bed fibrosis and inflammatory changes consistent with pathological complete response (Fig. 5) [11]. At follow-up, the patient reported high satisfaction with the cosmetic result and had no functional limitations.

## Discussion

Endoscopic nipple-sparing mastectomy addresses aesthetic concerns associated with traditional open mastectomy by concealing incisions and providing enhanced visualization for precise dissection. Meta-analyses have demonstrated that nipple-sparing mastectomy, when performed in appropriately selected patients, offers oncologic outcomes comparable to conventional mastectomy [11]. In HER2-positive breast cancer, high rates of pathological complete response following neoadjuvant chemotherapy increase eligibility for nipple preservation without compromising oncologic safety [9]. Immediate reconstruction with a DIEP flap complements E-NSM by restoring breast volume with autologous tissue whilst minimizing donor-site morbidity [6, 7]. Despite these advantages, adoption of E-NSM in resource-limited settings is constrained by the learning curve, equipment costs, and the need for combined expertise in endoscopic and microvascular techniques [12, 13]. Recent studies have demonstrated the feasibility of endoscopic nipple-sparing mastectomy combined with implant-based reconstruction as well as latissimus dorsi flap reconstruction, reporting favourable aesthetic outcomes with minimal visible scarring [14, 15]. However, these approaches are largely limited to prosthetic or pedicled reconstructions and may not provide the long-term advantages associated with microsurgical autologous tissue transfer.

From a broader perspective, this case is notable as it demonstrates the integration of advanced endoscopic breast surgery with microvascular autologous reconstruction following neoadjuvant chemotherapy in a resource-conscious setting. Whilst such combined approaches are increasingly reported from high-volume centres in East Asia and Western countries, published experience from low- and middle-income regions remains limited. The successful outcome in this patient underscores that, with appropriate patient selection, multidisciplinary collaboration, and institutional expertise, E-NSM with immediate DIEP flap reconstruction can be safely implemented beyond traditional high-resource environments.

## Conclusion

Endoscopic nipple-sparing mastectomy with immediate DIEP flap reconstruction following neoadjuvant chemotherapy is feasible and safe in carefully selected patients. With appropriate expertise and patient selection, this technique can achieve oncologic adequacy whilst maximizing aesthetic benefit.

## Data Availability

All relevant clinical data are included within the manuscript.

## Patient perspective

The patient expressed satisfaction with the surgical outcome, particularly appreciating the absence of visible breast scars and the natural appearance of the reconstructed breast.
